# Packaged water: optimizing local processes for sustainable water delivery in developing nations

**DOI:** 10.1186/1744-8603-7-24

**Published:** 2011-07-29

**Authors:** Ayokunle C Dada

**Affiliations:** 1 Institute of Ecology and Environmental Studies, Obafemi Awolowo University, Ile-Ife, Nigeria

## Abstract

With so much global attention and commitment towards making the Water and Sanitation targets of the Millennium Development Goals (MDGs) a reality, available figures seem to speak on the contrary as they reveal a large disparity between the expected and what currently obtains especially in developing countries. As studies have shown that the standard industrialized world model for delivery of safe drinking water technology may not be affordable in much of the developing world, packaged water is suggested as a low cost, readily available alternative water provision that could help bridge the gap. Despite the established roles that this drinking water source plays in developing nations, its importance is however significantly underestimated, and the source considered unimproved going by 'international standards'. Rather than simply disqualifying water from this source, focus should be on identifying means of improvement. The need for intervening global communities and developmental organizations to learn from and build on the local processes that already operate in the developing world is also emphasized. Identifying packaged water case studies of some developing nations, the implication of a tenacious focus on imported policies, standards and regulatory approaches on drinking water access for residents of the developing world is also discussed.

## Background

The development and use of water portends wide-ranging implications for global survival, security, health and economic development [1]. This demands the need for water issues to be tackled at the highest political level. Consequently, enshrined in international covenants and attested to by world nation's heads are the MDGs, one of which is to halve the proportion of people without sustainable access to portable water and basic sanitation. Today, more than halfway into the deadlines, available figures reveal a large disparity between the expected and the achieved [2]. Following a general paraphrase of the paper in the first section, the gruesome challenges that make achievement of the Millennium Development Goals daunting task in the developing world is described in the second section of this paper. Given the prevailing social and technical cost needed to revitalize or put in place functional public institutions, associated technologies and political will power, it is much undoubted that the standard industrialized world model for delivery of safe drinking water technology may not be affordable in much of the developing world in the foreseeable future [3], the third section suggests packaged water as one of the low cost alternative water provision that could help bridge the gap. As presented in the fourth section of this paper, despite the established role that this drinking water source plays in developing economies and populations, its importance is significantly underestimated. The fifth section highlights a view point that promotes identification of means of improvement rather than disqualification of local provisions and processes in a bid to safeguard public health. Using relevant case studies, it also suggests possible implication of irrational adoption of global policies on water supply access for residents of the developing world. The sixth section concludes.

## The Daunting Challenge of Meeting the MDG targets

Global attention and efforts have been committed towards making the MDG target for water and Sanitation a reality. It is more than halfway into the deadline, yet available figures seem to speak on the contrary especially in the developing world [2]. In most regions of the world, this target is far off-track [2,4,5]. Reports have shown that going by current trend, the global MDG sanitation target will be missed by more than half a billion people if the trend 1990-2004 continues up to 2015 [2]. A recent report asserts that the potential challenges to actualizing the MDGs are basically: maintaining the gains already made, coping with a rapid pace of urbanization, and a huge backlog of unserved rural people [6]. Although, some regions will reach the drinking water and sanitation target, places like sub-Saharan Africa remain an area of greatest concern. For example, in sub-Saharan Africa, with an 85% increase in urban population from 1990 to 2004, the number of urban dwellers unserved with either safe drinking water or basic sanitation doubled from 1990 to 2004 [6]. Recent studies suggest that in addition to rapid urbanization [7], ineffective governance ([8,9]) and persistent poverty [10] remain the root cause of water infrastructure associated problems that might make the goal far from being realistic in developing nations.

A recent article [11] presents thought provoking insights to the debate on the achievement of the MDGs. Optimistic as the millennium development targets may sound, some debates are however evident. It may be argued that there are fundamental problems associated with the statement of these goals and the means of measuring progress towards meeting them. There are wide definitional variations of what constitutes "safe drinking water" and "basic sanitation". These variables are difficult to measure and each has widely different cost and effort implications [11]. Thus, indicators chosen for monitoring the MDGs are often confusing, misrepresenting and many a times missing. Examples abound to attest to this. In India, a household is considered to have access if there is a water source within one mile (1.6 km) and in many cases; it is not the individual or the household access that is measured but the village as a whole. In Lagos, the newest major mega city in Africa with so many slums and shanty towns [12], the case could be different. What a kilometre distance means to urban dwellers in Lagos would differ significantly to the structures and population densities that are available in the rural settlements, yet they apparently have the same indicator yardstick. Currently based on existing data sources access is often taken to be a facility such as a standpipe, well, or public toilet within reasonable distance [11]. Again, masking effects of richer populations being served over unserved on the overall figures presented might be evident. This could stand as potent demodulators for aggressive and timely interventions for deprived residents in deficient locations.

Even where there is a water source it may not necessarily be accessible to all owing to other associated physical, economic or social complexities [11]. Apart from distance, the cost, level of sharing and queuing are decisive factors that determine actual water availability, accessibility and use [13,14]. Again, in practical terms, it is not clear what providing "basic" amenities will actually mean, and this will most likely vary in different contexts and countries [11]. Furthermore, access to the 'improved access' might not necessarily infer adequacy for a healthy life as access to water within a kilometre's reach may not be convenient or sufficient to protect health. Optimally, water should be made available at home [15] or at least within a hundred meters or five minutes total collection time, which has been observed to make a difference with regard to actual water use [16,17].

## Packaged Water: A Local Drinking Water Initiative

**Figure 1:**
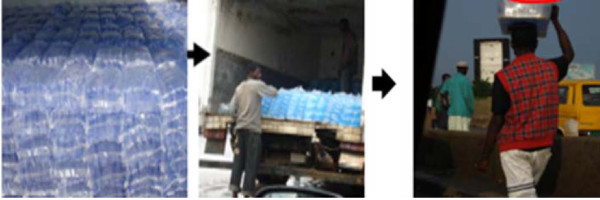
**Roadside vendors hawking chilled water in sachets**.

Several water supply models are already established, tested and proven effective in the developed world. Given the prevailing social and technical cost needed to revitalize or put in place functional public institutions, associated technologies and political will power, it is much undoubted that the standard industrialized world model for delivery of safe drinking water technology may not be affordable in much of the developing world in the foreseeable future [3], Subsequently, with the renewed global commitments towards the MDGs marked for 2015, the importance of locally sourced, low-cost alternative drinking water schemes in contributing to increased sustainable access in rural and peri-urban settings of developing nations cannot be over-emphasized [18]. One of such local interventions in Nigeria, where public drinking water supply is endemic is packaged drinking water [19]. This form of packaged water is usually distributed and sold in sachets (Figure 1). Packaged water refers to water that is packaged generally for consumption in a range of vessels including cans, laminated boxes, glass, plastic sachets and pouches, and as ice prepared for consumption [20,21].

Scattered around the breadth of developing nations are small, medium and large scale industries that manufacture packaged water sold as sachets (commonly referred to as pure water). The package water industry started initially as a cottage business to meet the demand of the thirsty population not adequately catered for by the available municipals. Today, the packaged water industry has become part of the unofficial economy as the sales of thousands of brands of thermoelectrically sealed nylon sachets containing about 0.5 L water have increased tremendously in many developing nations. Sold by the poor and patronised by members of the low and middle socio-economic class, this form of water started out as 'iced water' which was simply hand-tied nylon pouches containing treated or untreated cold water. Treatment then was simply with the use of absorbent pads (referred to as 'foam'), although it was thought to trap the all dirt and germs, it was largely effective for removal of suspended solids. Recent studies [3,22] confirmed the persistence of this drinking water source in some parts of Ghana owing to its affordability.

**Figure 2:**
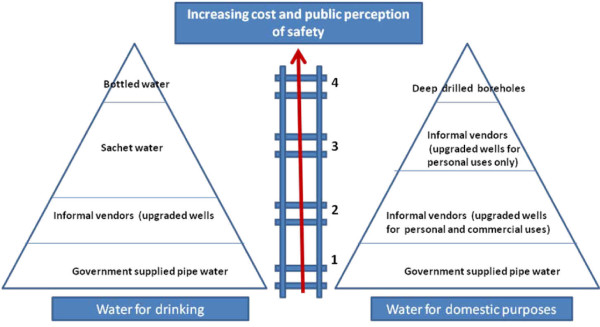
**Water for drinking and domestic purposes: Nigerian Water Supply Model**.

**Figure 3:**
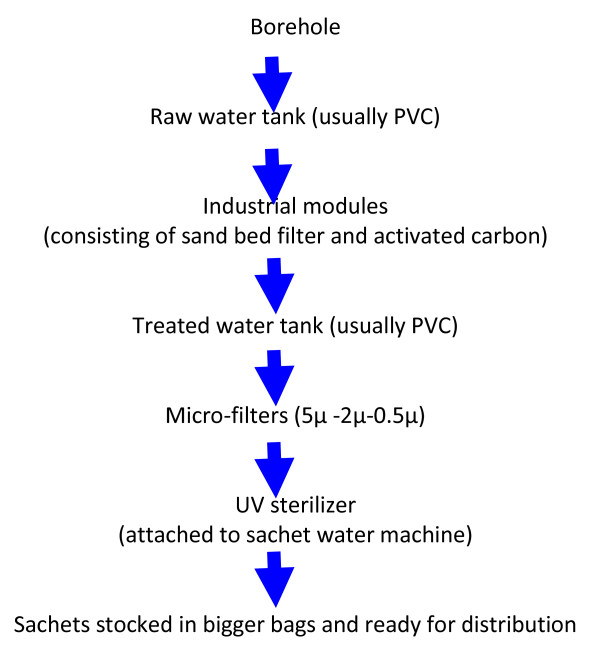
**Water treatment process in a typical sachet water factory**.

In urban Tanzania, a similar experience prevails based on the result from a recent survey that showed a significantly higher proportion of the population depend on packaged water owing to its perceived safety as compared to water from public pipes [23]. The hierarchical order of perception of safety in Nigerian water supply model is presented in Figure 2. The public perception safety in favour of packaged water in Nigeria stems out partly from the inadequate attempts of previous governments to provide potable piped water. The second contributing factor to this perception is the prevalent doubt on the quality of 'piped water' supplied at a reasonable charge by many informal vendors at the community level. The lack of trust in the quality of water supplied by these informal vendors is attributable to the subjectivity in the construction of wells from which the water is outsourced. There is currently no formal abstraction management or regulatory scheme in place in the entire nation. The effect of rapid urbanization in cities like Lagos has seen the sprawling city extending far beyond its original lagoon setting to encompass a vast expanse of mostly low-rise developments including as many as 200 different slums where living conditions are extremely crowded and dismal [12]. The resultant effect is a continuous reduction in the spacing between inter- and intra-building septic tanks and water wells, thus increasing the vulnerability of available sources to pollution from anthropogenic influence. Yet, anyone at any point in time with the necessary financial wherewithal could employ the service of cheap local labour to dig wells of subjective depths and specifications for water vending purposes. In most instances, as a cost saving measure, only a few concrete well rings are fixed to support the dug well (Figure 3).

Water pumped from these 'upgraded' wells into elevated storage tanks are widely referred o as 'borehole water' or 'tap water' among the nation's populace. It costs about N100,000 (about USD800) to put this system in place. On the other hand, to install a deep drilling system using heavy duty boring machines require a minimum of N400,000 (about USD3,200). In most instances, only a restricted proportion of the citizenry, precisely the wealthy, can afford the expensive deep drilling that conventional borehole technologies offer. In most instances, when this is the case, the water is strictly for owner usage and is not available for commercial purposes. Despite the debates associated with the quality of water provided by these upgraded wells of informal vendors, water from such sources is cheap (costs about 20-50 cents for 50 Litres), readily available but usage is only restricted for domestic uses alone - washing, bathing and cleaning. Sachet water, costing 50 cents for 3 Litres (one bag containing 20 sachets each of 150 ml volume), is thus often relied upon for drinking purposes. Although restively more expensive than water for domestic uses sourced from upgraded wells of informal vendors at the community level, a public perception of safety prevails - *at least it must have gone through one form of treatment or the other, even if they were gotten from questionable sources *[24].

Realistically sachet water produced in recent years by small-scale industries has experienced drastic improvement as the raw water is now treated by aeration, double or single filtration using porcelain molecular candle filters or membrane filters (Figure 3) and in some instances, disinfection is applied. The level of treatment generally depends on the source of water [3]. Although there are still inherent quality and regulatory challenges associated with this form of drinking water [25], the importance of this form of drinking water is well acknowledged by civil societies and nation governments of the developing world [26].

## Seeking Solutions For Local Problems: Local Processes Or Global Policies?

Despite the established role that this drinking water source plays in developing economies and populations, its importance is however significantly underestimated or not appreciated as presented by currently available 'guidelines' and 'policies' of internationally respected organizations - most of which are irrationally copied and adopted by the developing world [27]. The denial process stems from the equalisation of reasons attached to people's patronage of packaged water for the developing and the developed world. For example, a report by NDRC [28] asserts that given the explosion in bottled water use in the United States, driven in large measure by marketing designed to convince the public of bottled water's purity and safety, and capitalizing on public concern about tap water quality, people are willing to spend from 240 to over 10,000 times more per gallon for packaged water than they typically do for tap water [28]. As analysed by Riemann and Bank [29] in their study on regulatory standards for the developed and developing world, this is characteristic of a typical 'risk-averse' western world. Factors that stimulate demand in the developing nations are apparently different. In many developing nations, contamination of municipal water supply systems by faecal bacterial pathogens has become a public health hazard, because often there are not enough resources (cost, capital or commitment) to install or to operate functional municipal water systems leaving the civil society to resort to alternatives, one of which is packaged water.

In a similar vein, a World Health Organization (WHO) report [30] also itemizes the reasons for global consumption of packaged water. However, not elucidated in this report is the fact that packaged drinking water is an alternative source and its patronage, more of a survival strategy for many residents of the developing world rather than the reasons mentioned. This typifies the international community's partial or non-recognition given to this drinking water initiative which of course is a social adaptation to failed public municipals in the developing world. England felt a similar wave of the heat when the 2007 summer floods hit Gloucestershire leaving thousands of people to survive on packaged water which was generously distributed by the government and intervening organizations as relief aid [31]. It is much undoubted that what classifies patronage of packaged water as a survival strategy, psycho-satisfaction or a mere show of socio-economic status is more or less subjective differing with the particular prevailing situation.

In addition to the wrongly perceived cause and effect hypothesis, another conflicting view point is the definitional classification using certain 'exclusion criteria'. This is typified in the WHO report [30] that refers to 'bottled water' and 'packaged water' interchangeably without making concrete distinction between the two terms (for instance, Section 6.5.2: p114). In the report, households that relied on packaged water along with other vended sources are classified using the 'exclusion criteria' as not having 'reasonable access' to improved water supply along with those who get theirs from unimproved wells or surface water sources (Additional file 1, Table S1). It should however be noted that water obtained from these recommended 'improved sources' can also have a significant increase in contamination between the source and storage. A report [16] suggested possible contamination arising from two distinct physical domains- the public (outside the household) and the domestic (inside the household). Thus it may be realistic to suggest that the proportion of the world's population actually using safe drinking water is likely to be lower than that using the globally recognized 'improved' drinking water sources.

**Figure 4:**
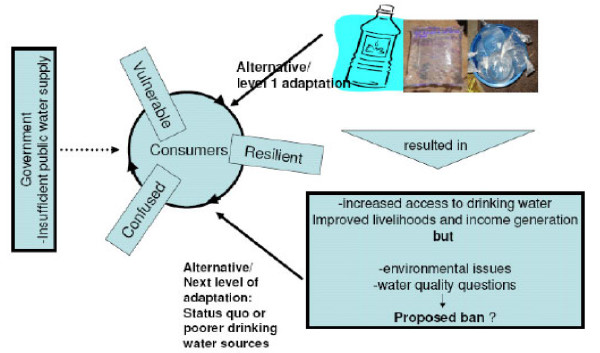
**Fate of the Public with irrational regulatory policies**.

Going by the 'exclusion criteria' and the 'official indicators', progress towards the water target of the MDGs is achieved as people switch to piped water connections, or to free public stand pipes, boreholes, or rainwater cisterns within a kilometre of their home [10,32,33]. But the daunting challenge is the time frame for which this could become a reality for residents of the developing. Apparently, it appears not to be too realistic a goal in the near future given the insufficiency of capital, cost (operation and maintenance) and commitment evident in most rural and urban settlements of low and medium income countries where municipal water supply functions are sub-optimal. As presented by the WHO report and other available literature, bottled water is considered unimproved and sachet water or other forms of packaged water are nowhere to be found either in the identified classes. By oppressing packaged water in a bid to protecting public health in developing nations, there is a danger that authorities could be making it still more difficult for deprived residents to obtain water which again could lead to more grievous conditions as people may revert to poorer sources (Figure 4). Agreeably, proffered recommendation to improving drinking water access in developing nations may not simply be about disregarding packaged water or other local initiative as unimproved. Instead, questions need be raised about what could be done to increase the effectiveness of the treatment and distribution system and how it could ultimately make a positive contribution to the widely publicised MDGs. It is therefore logical to conclude that there may be cases where improving services from so-called 'unacceptable options' (local provisions) can make much more significant difference to the well-being of the most deprived populations than striving for ideal solutions such as universal piped water connections [33].

## Implication for Policy and Way Forward

The developing world is masked with challenges of coping with failing infrastructures, inadequate finance, poor legislation, lack of appropriate institutional capacity for regulation and control and often the political will to enforce control measures. The position is complicated by the fact that many of these developing nations are at a loss on how to set standards [27]. Consequently, they resort to dependence on adopted standards, policies and guidelines as presented by international organizations based on scenarios and context in the developed world. These are only moderately modified and ultimately imbibed into national regulatory structures of developing nations without the means of attainment. There could be possible serious implication of a tenacious focus on such policies, standards and regulatory approaches imported from developed countries on drinking water access for residents of the developing world (Figure 1.1) as each situation differs in its own respect and has to be treated as such. For example, the WHO bacteriological water guidelines are widely accepted in industrialized as well as developing countries but they are not always achieved in practice [34].

Solving the water-related problems in the unserved regions of the developing world will demand acknowledgement, and attendant support of local processes that already exist in such locations. This will ultimately enable the local entrepreneurs to positively contribute their little yet significant quota to the achievement of international goals. Arguably, as a DFID report [35] portrays it, the best way to meet the people's demand for clean water and sanitation is to work with civil societies and government to help enable the voices of those without access to be heard and then for the governments to act on what they hear. In the past, many attempts by service providers and intervening non-governmental agencies have one way or the other failed because they allegedly did not involve the local community and already existent local processes in their action plans. Case studies are presented shortly of some on-going success stories in the packaged water industry. In the cited locations, various levels of stakeholder participation led to the birth of solutions that found a right balancing in between safeguarding public health through enactment of regulatory standards and improving social welfare through sustained access to packaged drinking water.

## Lagos, Nigeria

An alternative to erratic pipe borne drinking water supplies in Nigeria is Sachet water, popularly referred to as 'pure water'. A high demand drinking water alternative, it is sold by the poor and finds patronage from members of low and middle socio-economic class [36]. With concerns of questionable quality of packaged drinking water, the national regulator, NAFDAC, declared a 'gradual' nationwide ban on sachet waters to allow the manufacturers of sachet water to start winding-up or change to bottle packaging [26] which is more expensive but perceived safer than water in sachets. Successful implementation of this ban on sachet water has remained far from reality as a vast majority of its population depends on it. Today, the sachet water market is witnessing tremendous growth. In a recent survey [24] of thirty-four households in the location, 86.5% of respondents claim that they cannot cope if the proposed ban were to hold given the unavailability of other affordable drinking water options. A total of 73.5% claim increased price associated with the bottled form of packaged water will ultimately deny them access to the suggested option by the regulator. It became obvious that the most probable outcome of such a ban of sachet form of packaged water is a reversion to poorer sources. Again, the federal government did assert that sachet water industry has remained one of the most successful poverty alleviation programs it embarked upon since independence. The proposed ban was consequently suspended. Focus was redirected to other means of improving the sachet water industry to produce the desired results and ultimately safeguard public health.

## India

The late nineties marked the commencement of packaged drinking water regulation in India. Solely handled by the Bureau of Indian Standards in collaboration with the Health Ministry, the rules on its safety were drafted into a Prevention of Food Adulteration Act. The original plan was to come up with a standard that matches with international standards. Given the complexities and the technologies involved in the implementation, the PFA Act however remained vague on the issue of allowable levels of pesticides in packaged drinking water. With growing health concerns, a stakeholder meeting between the BIS and the Health Ministry officials marked the declaration of specific allowable limit - no pesticide should exceed 0.0001 mg/litre and total content of pesticide not exceeding 0.005 mg/litre. It was agreed that testing methods and support are to be provided by the BIS. Again, consensus was reached that it will take some time before the necessary changes take effect in the packaged water industry [37].

## Accra, Ghana

A rapidly emerging water vending business in Ghana has been that of vending sachet water or bagged water, which is a cheaper alternative to bottled water [22]. Given the unreliable supply of drinking water by the municipals, a large proportion of the people depend on this bagged form of drinking. To ensure that Ghanaians have access to cheap clean water, sachets approved by the Ghanaian government that meet the Ghana Standards Boards sanitation requirements, are being sold all over the country [38]. These sachets are one of the main contributors to the trash that blankets the streets and gutters. The Accra Metropolitan Assembly (AMA) declared a possible ban on the use of sachet and polythene bags for drinking water, if manufacturers of such products refused to immediately negotiate with the AMA on concrete proposals as to how to deal with the menace of plastic waste [39]. This led to the birth of several stakeholder forums that heralded the emergence of a recycling taskforce - people picked from the government, plastic manufacturers, water sachet producers and city authorities to encourage and facilitate the recycling of the sachets, creation of new recycling plants as well as working with existing recyclers to expand their facilities [40]. Sponsored by some sachet bag manufacturers, several campaigns on plastic waste disposal and management were launched at Primary and Junior Secondary Schools in the nation to educate the school children on the proper disposal of plastic waste and how to manage such waste. In addition to this, an Accra based NGO, focusing on sustainability and the environment has taken on the task of cleaning up the streets of Accra. They employ staffs which does not include the local Ghanaians that are compensated for collecting the sachets. To date, this NGO has taken over 10 million sachets off the streets of Accra [38], the proposed ban on sachet water was suspended and residents still have access to drinking water in sachets.

As presented by the examined case studies, it is evident that rather than simply disqualifying packaged water as portrayed in respected international literature, focus should be on identifying means of improvement. For instance, more research could be conducted with focus placed on determinants of the final quality of produced sachet water - treatment, handling and distribution practices. Furthermore, international and local organizations and national health agencies could help facilitate research targeted at the identification, substantiation and incorporation of Hazard Analysis Critical Control Points (HACCP) and limits. This would address hazard analysis of the treatment and distribution processes and ultimately herald the emergence of a workable water safety plan that applies specifically to the packaged water industry. Intensified efforts on local and international research that major on the production of readily accessible and adaptable in-house water testing kits could also play a significant role in fortifying daily in-house bacteriological monitoring of the finished products. International organizations could also partner with regulatory agencies and civil societies to facilitate intensive hygiene and sanitation awareness training programs for vendors, manufacturers and other relevant stakeholders in the packaged water industry. Predictably, the implementation of these will yield desired results that would warrant a better packaged water industry, an improved social welfare through sustained access to drinking water and ultimately, a safer public at large in the developing world.

## Conclusion

Packaged water made available in sachets, like other local initiatives offer substantial hope in contributing to increased sustainable access in rural and urban settings of developing nations if acknowledged and improved upon. The call is therefore made to intervening global communities and developmental organizations for the need for to learn from and build on the local processes that already operate in the developing world. Room for optimum improvement, via collaborative efforts with relevant stakeholders will demand striking a suitable balance between two preferred options: promoting public health (through improved regulation of the packaged water industry) while concurrently improving social welfare (encouraging access through support of these initiatives that cover for institutional inadequacies in public water supply coverage).

## Competing interests

The author declares that they have no competing interests.

## Consent

Written informed consent was obtained for publication of the accompanying images.

## Supplementary Material

Additional file 1 **Table S1**: Definition of access to water [30]. Click here for file
