# Supplementary material for: Packaged water: optimizing local processes for sustainable water delivery in developing nations
Source: Global Health. 2011 Jul 29;7:24. doi: 10.1186/1744-8603-7-24 (PMC3161851; doi:10.1186/1744-8603-7-24)
Supplement: Additional file 1 — Table S1: Definition of access to water [30]. [file 1744-8603-7-24-S1.DOC]

| **Improved water supply technologies** | **Unimproved water supply technologies** |
| --- | --- |
| —Household connection, Public standpipe  —Borehole, Protected dug well  —Protected spring, Rainwater collection | —Unprotected well, Unprotected spring  —Vendor-provided water  —Bottled water  —Tanker truck provision of water. |
